# Motor unit potential morphology differences in individuals with non-specific arm pain and lateral epicondylitis

**DOI:** 10.1186/1743-0003-5-34

**Published:** 2008-12-16

**Authors:** Kristina M Calder, Daniel W Stashuk, Linda McLean

**Affiliations:** 1 School of Rehabilitation Therapy, Louise D. Acton Building, 31 George Street, Queen's University, Kingston, Ontario, Canada; 2 Department of Systems Design Engineering, University of Waterloo, Waterloo, Ontario, Canada

## Abstract

**Background:**

The pathophysiology of non-specific arm pain (NSAP) is unclear and the diagnosis is made by excluding other specific upper limb pathologies, such as lateral epicondylitis or cervical radiculopathy. The purpose of this study was to determine: (i) if the quantitative parameters related to motor unit potential morphology and/or motor unit firing patterns derived from electromyographic (EMG) signals detected from an affected muscle of patients with NSAP are different from those detected in the same muscle of individuals with lateral epicondylitis (LE) and/or control subjects and (ii) if the quantitative EMG parameters suggest that the underlying pathophysiology in NSAP is either myopathic or neuropathic in nature.

**Methods:**

Sixteen subjects with NSAP, 11 subjects with LE, eight subjects deemed to be at-risk for developing a repetitive strain injury, and 37 control subjects participated. A quantitative electromyography evaluation was completed using decomposition-based quantitative electromyography (DQEMG). Needle- and surface-detected EMG signals were collected during low-level isometric contractions of the extensor carpi radialis brevis (ECRB) muscle. DQEMG was used to extract needle-detected motor unit potential trains (MUPTs), and needle-detected motor unit potential (MUP) and surface detected motor unit potential (SMUP) morphology and motor unit (MU) firing rates were compared among the four groups using one-way analysis of variance (ANOVA). Post hoc analyses were performed using Tukey's pairwise comparisons.

**Results:**

Significant group differences were found for all MUP variables and for MU firing rate (*p* < 0.006). The post-hoc analyses revealed that patients with NSAP had smaller MUP amplitude and SMUP amplitude and area compared to the control and LE groups (*p *< 0.006). MUP duration and AAR values were significantly larger in the NSAP, LE and at-risk groups compared to the control group (*p *< 0.006); while MUP amplitude, duration and AAR values were smaller in the NSAP compared to the LE group. SMUP duration was significantly shorter in the NSAP group compared to the control group (*p *< 0.006). NSAP, LE and at-risk subjects had lower mean MU firing rates than the control subjects (*p *< 0.006).

**Conclusion:**

The size-related parameters suggest that the NSAP group had significantly smaller MUPs and SMUPs than the control and LE subjects. Smaller MUPs and SMUPs may be indicative of muscle fiber atrophy and/or loss. A prospective study is needed to confirm any causal relationship between smaller MUPs and SMUPs and NSAP as found in this work.

## Background

Long-standing static contractions or repetitive work, such as that performed in computer and assembly line environments, can lead to chronic muscle pain [1-3]. The wrist extensor muscles have been implicated in a condition called non-specific arm pain (NSAP) or work-related upper limb disorder, which, as the names suggest, has an unknown pathophysiology. Patients with NSAP complain of diffuse forearm pain during and after tasks that require repetitive wrist motion, and they have muscle pain and tenderness on palpation that is not consistent with lateral epicondylitis (LE), a known tendinopathy resulting from repetitive wrist extension. The similarity between LE and NSAP is that the pain radiates down the forearm and can be replicated during resisted movements of the extensor muscles of the wrist during work. In NSAP, the signs and symptoms include diffuse pain in the forearm after aggravating activities, muscle tenderness to palpation, reduced grip strength, and functional loss [4]. A diagnosis of NSAP is made when there is an absence of objective clinical signs associated with known upper limb disorders, such as medial or lateral epicondylitis, deQuervain's tendonitis and cervical radiculopathy [4]. Although there appears to be agreement on the mechanical factors that lead to the development of NSAP (i.e., repetitive movements and sustained postures), there are conflicting views regarding the underlying pathophysiology of this condition [5]. Because we do not know what structures are affected and in what way, we cannot properly diagnose or treat this form of repetitive strain injury.

Some authors believe that chronic pain conditions such as NSAP and trapezius myalgia, are associated with damage within the muscle [1,6-10], whereas others believe they are caused by neuropathic changes [11-14]. Patients with chronic trapezius myalgia related to static muscle loading during assembly work have been the focus of several studies where biopsies of the descending portion of the trapezius muscle have been taken in an attempt to determine the underlying pathophysiology. 'Ragged-red' fibers have been identified in these samples, and researchers have therefore suggested that the origin of this condition is associated with mitochondrial damage to the Type I fibers [1,6,8], however, these results have not been conclusive, with similar damage noted in individuals who perform similar tasks but who are pain free. Dennet et al, [10] took specimens from the first dorsal interosseous muscle of keyboard operators with chronic overuse syndrome and, when compared to healthy controls, found an increase in the number of type I fibers, a lower number of type II fibers, type II fiber hypertrophy, and mitochondrial changes. Other researchers have found indications that chronic muscle pain in the wrist flexor group (also referred to as NSAP) may be neuropathic in nature [11,13]. In particular, Greening et al. speculate that NSAP affecting the wrist flexor muscles is neuropathic in origin based on observed changes in median nerve function [11,12,15].

In the current study, we have defined NSAP as a condition affecting the wrist extensor muscles. These muscles are innervated by the radial nerve, and therefore differential diagnosis would include radial neuropathy, cervical radiculopathy (C6) and muscle or tendon pathology. To our knowledge, no evaluation methods, including electromyographic (EMG) analysis techniques, have been used to characterize muscles affected with NSAP. Changes in motor unit morphology or firing pattern characteristics may indicate the underlying pathophysiology associated with the contractile deficits seen in this population.

Shape characteristics of motor unit potentials (MUPs) provide insight into the underlying pathophysiology of neuromuscular diseases [16-18]. In myopathies, classic EMG findings are MUPs with reduced durations and amplitudes due to loss of muscle fibers or fibrosis [17]. There is also increased complexity in the MUP waveforms, which may be associated with atrophic or regenerating muscle fibers [19], or with temporal dispersion among muscle fiber potentials due to fiber diameter variations [17]. In neuropathies, classic EMG findings include increases in MUP duration and amplitude caused by increased fiber number and density as orphaned muscle fibers receive axonal sprouts from healthy axons. In this case, the number of turns and phases may either be normal or increased [17].

In the present study, EMG signal decomposition-based algorithms and quantitative MUP analysis techniques were used to investigate the electrophysiological characteristics of motor units (MUs) in healthy control subjects, subjects deemed at risk for developing a repetitive strain injury (RSI), and in individuals with LE and NSAP. The purpose of this study was to determine: (i) if MU morphology and firing pattern statistics as represented by quantitative parameters of MUPs detected in an affected muscle in individuals with NSAP are different from those with LE and/or control subjects and (ii) if these quantitative EMG parameters suggest that the underlying pathophysiology in NSAP is either myopathic or neuropathic in nature.

## Methods

### Subjects

A four-group cohort design was used in this study. Subject recruitment occurred in the Kingston community by advertisements posted in the local newspaper and posters placed in local physiotherapy clinics and physicians' offices. The advertisements asked for volunteers to participate if they were between the ages of 18–60 years and either (i) experienced elbow or forearm pain attributed to their repetitive work/activities; (ii) worked in a repetitive job where their colleagues were developing repetitive strain injuries, but they had no symptoms themselves; or (iii) did not work in a job that required repetitive hand movement or perform activities (sports/arts) that were repetitive (e.g., tennis/guitar) and had no current or previous upper limb injury or pain. Potential subjects underwent a telephone screening interview to ensure that they met the inclusion and exclusion criteria. Subjects were excluded at the time of the interview if their symptoms suggested that they might have a concurrent cervical pathology; if their pain originated at the wrist, hand or anterior forearm; or if they had a history of heart disease, lung disease, neurological conditions or diabetes. The study was approved by the Queen's University Health Sciences Research Ethics Board (REH-183-03), and all subjects provided written informed consent prior to participation.

### Experimental procedures

A clinical examination was performed for demographic comparison among the groups who were exposed to repetitive tasks (NSAP, LE, and at-risk subjects), to verify correct group assignment and to verify that subjects had no signs or symptoms of cervical radiculopathy and/or other repetitive strain injury, such as carpal tunnel syndrome, deQuervain's tendonitis, or medial epicondylitis. The screening examination consisted of a neurologic examination of the upper extremities, including myotome testing, dermatome (light touch, pin prick) testing, and assessment of the deep tendon reflexes at the C5 to C8 levels. Cervical spine range of motion was tested in sitting to ensure that cervical movements did not reproduce the forearm symptoms. The movements tested included flexion, extension, lateral flexion, rotation, and combined extension with lateral flexion. These movements were held at the end of the available range of motion for 10 seconds. Three repetitions of maximal handgrip strength (Jamar Dynamomter, Sammons Preston Inc., Model # 5030J1; in position 2) and maximal pinch grip strength (Baseline Evaluation Instruments, 60# mechanical pinch gauge, model # 12-0201) were measured bilaterally with the elbow flexed to 90 degrees, and with the wrist held in neutral between flexion and extension, respectively.

A pressure algometer (model PTH-AF 2 Pain Diagnostic and Treatment Corporation, Great Neck, NY 11021, USA) was used to measure pain pressure threshold (PPTh) and pain tolerance (PPtol). The device consists of an analog force gauge fitted with a disc-shaped rubber tip (1 cm^2^). The range of the gauge is 0–10 kg, with increment markings at 0.1 kg. Measurements were made at the nail bed of the third digit (D3) and over all the bellies of the extensor carpi radialis brevis (ECRB) muscle, the flexor carpi radialis (FCR) muscle, the biceps brachii (BB) muscle and the triceps brachii (TB) muscle. Pain tolerance scores (PPtol) were normalized to the amount of pressure subjects could withstand being applied to the nail bed on D3 of the affected (or tested) limb.

Subjects were assigned to the LE group if their symptoms were reproduced with resisted wrist extension in neutral, passive wrist flexion with the elbow extended and if there was pain on palpation of the lateral epicondyle. This resisted wrist extension test was performed by stabilizing the subject's forearm and then having him or her form a fist and actively extend the wrist, keeping the elbow fully extended. The examiner then attempted to force the wrist into flexion. A reproduction of the subject's pain at the lateral epicondyle during this contraction was considered a positive test [20]. Subjects in the LE group experienced pain on palpation of their lateral epicondyle. If pain occurred during palpation of the ECRB muscle in subjects in the LE group, it could not be reproduced by palpating at a distance greater than 3 cm distal to the cubital crease [4] since pain that is experienced more distally on the wrist extensor group might be due to muscle tenderness within the ECRB itself and not exclusively at the tendon.

Subjects who were assigned to the NSAP group experienced pain on palpation of the ECRB muscle and complained of forearm pain during wrist extension activities performed at work or in their leisure activities, but resisted wrist extension with elbow extension (as described above) did not reproduce their signs and symptoms. Because our goal was to characterize individuals with NSAP separately from LE, we did not include any subjects who had signs or symptoms that could be attributed to both LE and NSAP. At-risk subjects had no pain on resisted wrist extension, passive wrist flexion, or palpation of the lateral epicondyle or the ECRB muscle. Subjects who were assigned to the asymptomatic at-risk group were required to have no history of arm injury or pain and to work in a job that demanded frequent or constant repetition of wrist extension, whereas the subjects in the control group did not perform repetitive wrist motions at work or during their leisure time.

Potential participants were excluded if resisted wrist flexion, passive wrist extension and palpation of the medial epicondyle reproduced symptoms, as these would indicate the presence of a wrist flexor pathology. The upper limb tension test (ULTT) with radial nerve bias (ULTT3) was performed as described in Kleinrensink et al. [21]. These tests were used for sample description only, not to rule out other pathology as they have questionable sensitivity and specificity [21,22]. Two self-report outcome measures, the Disability of the Arm, Shoulder and Hand (DASH) questionnaire [23] and the Short Form-36 (SF-36) questionnaire [24] were completed by the subjects. The DASH questionnaire measures the disability in persons with musculoskeletal disorders of the upper limb for both descriptive and evaluative purposes [23,25]; the 30-item questionnaire is scored out of 100, with a higher score indicating a greater disability. The SF-36 uses a 0–100 point scoring system that calculates health-related quality of life with eight health dimensions: physical functioning, role of physical functioning, bodily pain, general health, vitality, social functioning, emotional role, and mental health [24]. The control subjects did not undergo the thorough clinical evaluation performed on the other three groups since they were not in any pain and did not perform repetitive activities.

A total of 80 potential participants came to the laboratory for testing, and eight subjects who experience pain at their elbow and or wrist extensors were excluded following the clinical evaluation as they presented a mix of symptoms that did not meet the inclusion criteria for either the NSAP or LE group as outlined above, or that exhibited signs and symptoms of both NSAP and LE.

### Surface and intramuscular EMG recordings

**Figure 1:**
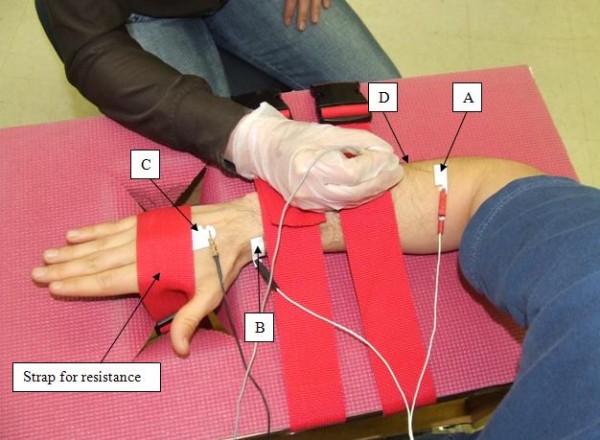
**Experimental set-up and electrode position**. The active electrode (A) was placed over the motor point of the ECRB muscle. The passive electrode was placed over the radial styloid process (B). The common reference electrode was placed on the dorsum of the hand (C). A concentric needle electrode (D) was inserted in a distal to proximal direction parallel to the muscle fibers so that the tip of the needle was underneath the active electrode (A).

All subjects underwent an electrophysiologic evaluation of the ECRB muscle. The affected limb or the more seriously affected limb (as determined by subjective complaint) was used for electrophysiologic evaluation in the LE and NSAP subjects. The dominant arm was selected in control subjects; for at-risk subjects, the limb selected (dominant/non-dominant) was matched to an LE or NSAP subject of the same age and sex. For this evaluation, subjects were seated in a straight-backed chair with the elbow of the tested arm flexed to 90° and the forearm pronated and resting on a custom-built table (Figure 1). Adjustable straps attached to the bottom of the testing table were passed through an opening and secured around the dorsum of the hand to provide resistance during the isometric extension contractions.

The DQEMG method and associated algorithms were used, as described in detail elsewhere [18,26]. Prior to electrode placement, the motor point of the ECRB muscle of the test limb was identified as the area over the muscle surface where the lowest possible electrical stimulus produced a minimal muscle twitch. The location of the motor point in the ECRB muscle was approximately two centimeters distal to the cubital crease. Using the cathode portion of a stimulating probe, with the train rate of the stimulator set at 10 pps and the stimulation duration set at 1 ms [27], the cathode was moved over the muscle belly until the motor point region was determined. The skin over the motor point, over the radial styloid process and over the dorsum of the hand of the test limb was cleaned with rubbing alcohol prior to electrode placement. A surface Ag-AgCl electrode (Kendall-LTP, Chicopee, Massachusetts) was cut in half to measure 1 cm by 3 cm. The active electrode was positioned over the motor point of the ECRB, and the reference electrode was placed over the radial styloid process to form a monopolar configuration. A full-sized surface electrode (2 cm by 3 cm) was positioned on the dorsum of the hand to act as the common reference. Subjects were asked to perform a 3 second maximum voluntary contraction (MVC) of their wrist extensors with verbal encouragement provided throughout. The peak root mean square (RMS) value calculated over contiguous one-second intervals of the surface EMG attained during the MVC was determined. This highest computed value represented the maximal voluntary effort (MVE) and all subsequent contractions were expressed as a percentage of the MVE and referred to as the %MVE-RMS.

A disposable concentric needle electrode (Model 740 38–45/N; Ambu^® ^Neuroline, Baltorpbakken, Ballerup, Denmark) was then inserted into the ECRB muscle using a distal to proximal approach so that the tip of the needle was approximately 2 cm deep underneath the active surface electrode. The needle position was adjusted until the average peak acceleration of the MUPs detected during a low-level contraction (5–10% MVE) was above 30 kV/s^2 ^[28]. Once a suitable needle position was found, the operator stabilized the needle manually and then asked the subject to hold a desired contraction force for 30 s. Subjects were provided with a visual bar graph and a numerical value that corresponded to their force output (%MVE-RMS) for feedback. Following each contraction, the needle was moved (medially, laterally, superficially and/or deeper) so that MUPs generated by a representative pool of motor units sampled from throughout the muscle would be detected. Each subject performed repeated contractions until at least 30 MUPTs were obtained. The contraction force varied between 5–20% of MVE. A 2-minute rest period was provided between contractions. AcquireEMG algorithms running on a Neuroscan Comperio EMG system (Neurosoft, Sterling, VA) were used to acquire the needle and surface EMG data during 30 s intervals with a sampling rate of 31250 and 3125 Hz respectively. The needle- and surface-detected signals were bandpass filtered from 10 Hz to 10 kHz and 5 Hz to 1 kHz respectively.

### Data reduction and analysis

After data collection, the MUPTs were evaluated through visual inspection. The acceptability of each MUPT, each needle-detected MUP, and each surface-detected MUP was based on previously reported criteria [26]. Briefly, an acceptable train had at least 50 MUPs and a consistent firing rate plot in the physiological range (8 Hz–30 Hz), as well as an inter-discharge interval (IDI) histogram with a Gaussian-shaped main peak and a coefficient of variation of ≤ 0.3 [29]. Any MUPTs that did not meet all of these criteria were excluded from analysis.

Markers indicating the onset, negative peak, positive peak and end of the MUP waveforms, and markers indicating the onset, negative peak onset, negative peak, positive peak, and end of the SMUP waveforms were automatically determined by the software; they were visually inspected for accuracy and manually repositioned if incorrectly placed.

Mean MU firing rate, and MUP and SMUP morphological parameters were measured and used as dependent variables in the data analysis. The MUP parameters included peak-to-peak amplitude, duration, number of phases, and area-to-amplitude ratio (AAR). The SMUP parameters included peak-to-peak amplitude, area, and duration.

### Statistical Analysis

Descriptive data are reported as means ± standard deviations, and were analyzed using MINITAB^® ^Statistical Software (v.14). Differences between group means for the demographic data (age, weight, height, disease duration, and wrist extensor MVC force) were tested using one-way analysis of variance (ANOVA) models. Demographic variables from the clinical evaluation were compared using one-way ANOVAs. The alpha level was set at 0.05 for all tests.

MUP and SMUP morphology and mean MU firing rates were compared among the four groups using one-way ANOVAs. The *α*-level was adjusted to account for multiple comparisons (*α *= 0.05/8) and was therefore set at *α *= 0.006. Post hoc analyses were performed using Tukey's pairwise comparisons.

## Results

### Demographic data

A total of 72 subjects participated: sixteen subjects with NSAP (7 men, 9 women), 11 subjects with LE (6 men, 5 women), 8 subjects at-risk (2 men, 6 women), and 37 control subjects (15 men, 22 women). The demographic data of the four groups are listed in Table 1. The subjects with NSAP had a mean symptom duration of 27(± 32) months, and the subjects with LE had a mean symptom duration of 39(± 31) months. There was no difference in this duration between these groups (*p *= 0.33). The control subjects were significantly younger in age and stronger in MVC wrist extensor strength than the other three groups (*p *< 0.05).

**Table 1:** Demographic data of the tested limb for the at-risk (*n *= 8), NSAP (*n *= 16), LE (*n *= 11) and control group (*n *= 37).

	**At-risk** **Mean ± SD**	**NSAP** **Mean ± SD**	**LE** **Mean ± SD**	**Control** **Mean ± SD**
Height (cm)	169.76 ± 9.20	165.58 ± 8.06	168.33 ± 7.63	171.62 ± 8.38
Weight (lbs)	148.38 ± 23.75	158.06 ± 29.74	165.91 ± 31.65	148.92 ± 24.17
Age (years)	44.75 ± 13.48**	50.25 ± 9.21**	46.55 ± 10.65**	27.09 ± 5.00*
MVC (N)	105.2 ± 52.18**	126.81 ± 50.89**	137.79 ± 57.29**	194.46 ± 51.30*

Parameters marked with * are significantly different from the other groups parameters marked with ** (*p *< 0.05)

### Clinical evaluation outcomes

The clinical evaluation measures from the at-risk, NSAP and LE group are shown in Table 2. The NSAP group had significantly higher DASH scores for all three modules than the at-risk subjects (*p *< 0.05). The LE subjects had significantly higher DASH scores for the disability module and sport/art module than the at-risk group (*p *< 0.05), but not the work module (*p *> 0.05). No significant differences in reported disability scores were found between the NSAP and LE groups (*p *> 0.05).

**Table 2:** Clinical evaluation outcomes from the Disability of arm shoulder and hand (DASH) questionnaire, SF-36 eight domain scores, ULTT3 (number of positive tests) pain threshold scores (values in brackets are normalized to third nail bed; D3), grip and pinch-grip strength for the at-risk, NSAP and LE groups.

	**At-risk**		**NSAP**		**LE**	
	**n**	**Mean ± SD**	**n**	**Mean ± SD**	**n**	**Mean ± SD**

DASH						
Disability score	8	0.66 ± 1.29*	16	23.83 ± 12.96**	11	32.39 ± 15.10**
Work module	8	0.00 ± 0.00*	15	35.22 ± 31.59**	11	28.98 ± 26.56
Sport/art module	4	0.00 ± 0.00*	9	68.06 ± 29.22**	7	56.3 ± 27.2**
SF-36						
Physical functioning	8	97.50 ± 3.78*	15	82.00 ± 18.01**	11	75.45 ± 17.39**
Role physical	8	100.00 ± 0.0*	16	62.50 ± 38.76**	11	50.00 ± 41.83**
Bodily pain	8	88.50 ± 10.35*	16	57.38 ± 18.75**	11	47.36 ± 21.72**
General health	8	84.88 ± 5.51	16	73.12 ± 20.04	11	68.27 ± 20.65
Vitality	8	73.75 ± 11.88*	16	61.56 ± 18.86	11	53.18 ± 19.01**
Social functioning	8	98.44 ± 4.42	16	84.38 ± 17.38	11	77.27 ± 31.03
Emotional role	8	83.33 ± 30.86	16	83.33 ± 32.20	11	81.82 ± 27.34
Mental health	8	88.00 ± 9.32	16	76.50 ± 16.58	11	73.09 ± 16.88
						
ULLT3 (n positive)	8	0	16	0	11	5
Pain Threshold (kg/cm^2^)						
D3	8	10.38 ± 5.87	16	12.87 ± 5.95	11	15.03 ± 6.23
ECRB	8	8.03 ± 4.82 (77%)*	16	5.78 ± 3.49 (45%)**	11	7.34 ± 4.16 (49%)
FCR	8	10.74 ± 6.73 (103%)	16	9.18 ± 5.06 (71%)	11	9.72 ± 4.86 (65%)
BB	8	8.48 ± 3.59 (82%)	16	9.08 ± 4.74 (71%)	11	10.65 ± 6.24 (71%)
TB	8	9.34 ± 4.72 (90%)*	16	8.28 ± 5.02 (64%)**	11	12.58 ± 6.72 (84%)
Grip strength (kg)	8	31.75 ± 5.56	16	33.95 ± 13.06	11	33.02 ± 12.53
Pinch-grip strength (kg)	8	7.39 ± 7.62	16	9.41 ± 3.89	11	11.14 ± 4.28

Parameters marked with * are significantly different from the other groups parameters marked with ** (*p *< 0.05)

Significant differences in four of the eight dimensions of the SF-36 were found between the three groups as shown in Table 2. The post hoc analysis revealed no significant differences between the NSAP and LE groups; however, significant differences were identified between the LE and at-risk individuals for the physical functioning, role physical functioning, bodily pain, and vitality dimensions (*p *< 0.05), where the individuals with LE scored significantly lower. The post hoc analysis also revealed that the NSAP group scored significantly lower than the at-risk subjects for the role of physical functioning and bodily pain dimensions (*p *< 0.05) and tended to score lower for the physical functioning dimension (*p *= 0.078).

The ULTT3 with radial bias revealed five out of 11 subjects had a positive test in the LE group, whereas none of the NSAP subjects or at-risk subjects had a positive test. The normalized score for PPtol on the ECRB and triceps brachii (TB) muscle was found to be significantly lower in the NSAP subjects compared to the at-risk subjects (*p *< 0.05). All other PPtol scores were not found to be significantly different among the groups (*p *> 0.05). Handgrip strength and pinch-grip strength of the tested limb were not significantly different among the groups (*p *> 0.05).

### MUP morphology and mean MU firing rates

Significant group differences were found for all MUP variables and for mean MU firing rate (*p *< 0.006) as identified in Table 3. Post hoc analyses revealed that the NSAP group had significantly smaller MUP amplitudes than the control and LE groups (*p *< 0.006). The at-risk subjects had MUP amplitudes that were not significantly different from any other group (*p *> 0.006). The control group had significantly shorter duration measures than the other groups, and the NSAP group had significantly smaller MUP durations than the LE and at-risk groups (*p *< 0.006). The NSAP group demonstrated greater number of phases than the control group (*p *< 0.006). Post hoc analysis of MUP AAR revealed that: i) AAR in the NSAP, LE and at-risk groups were all larger than the AAR of the control group and ii) the LE group had significantly larger MUP AAR than the NSAP and at-risk group (*p *< 0.006). Post hoc analysis of mean MU firing rate revealed significantly higher firing rates in the control group than the NSAP and LE groups (*p *< 0.006).

**Table 3:** Mean MUP and SMUP morphology and mean MU firing rates across the four groups.

	**Control** n = 37	**At-risk** n = 8	**LE** n = 11	**NSAP** n = 16
	Mean ± SD	Mean ± SD	Mean ± SD	Mean ± SD

**Needle-detected MUPs**				
Amplitude (μV)	491.1 ± 300.7*	444.1 ± 233.4	519.0 ± 426.6†	420.9 ± 282.4**‡
Duration (ms)	7.64 ± 2.85*	9.76 ± 2.99**‡	9.70 ± 3.16**‡	8.36 ± 2.68**†
Number of Phases	2.55 ± 0.71*	2.72 ± 0.77	2.63 ± 0.81	2.74 ± 0.82**
AAR (ms)	1.27 ± 0.43*	1.43 ± 0.39**▫	1.62 ± 0.59**†▫▫	1.34 ± 0.43**‡
				
Mean MU Firing rate (Hz)	14.98 ± 2.97*	14.73 ± 3.04	13.86 ± 2.71**	14.53 ± 2.68**
				
**Surface-detected MUPs**				
Amplitude (mV)	113.73 ± 90.73*	91.28 ± 49.45**	111.14 ± 109.53†	65.75 ± 41.58**‡
Area (mVms)	593.9 ± 507.4*	424.4 ± 230.1**	502.7 ± 518.7†	273.7 ± 182.6**‡
Duration (ms)	19.76 ± 5.52*	19.69 ± 4.54†	19.21 ± 5.93	17.25 ± 5.21**‡

Parameters marked with * are significantly different from the other groups parameters marked with ** (*p *< 0.006)

Parameters marked with † are significantly different from the other groups parameters marked with ‡ (*p *< 0.006)

Parameters marked with ▫ are significantly different from the other groups parameters marked with ▫▫ (*p *< 0.006)

### SMUP morphology

Significant group differences were found for all SMUP variables (*p *< 0.006), as identified in Table 3. The NSAP group had significantly lower SMUP amplitudes and areas compared to the control and LE groups (*p *< 0.006), and the at-risk group showed significantly lower SMUP amplitudes and areas compared to the control group (*p *< 0.006). The NSAP group had significantly shorter SMUP duration than the control and at-risk groups (*p *< 0.006).

## Discussion

Patients with NSAP present with inconclusive neurological and musculoskeletal system examinations even though they complain of debilitating pain during the performance of their work and activities of daily living. For this reason, the goals of this study were: i) to determine if quantitative EMG could be used to detect differences in neuromuscular physiology in a group of individuals with NSAP as compared to healthy subjects and ii) if such differences were identified, whether this condition was myopathic or neuropathic in nature. It is not until one knows what structures are involved that one can properly diagnose, treat and measure treatment effectiveness in this form of repetitive strain injury.

In the current study, individuals with NSAP and LE had higher disability scores and a decreased physical function when compared to the asymptomatic subjects using the DASH and SF-36 questionnaires. The DQEMG results identified significant group differences for all MUP variables and MU firing rate (*p *< 0.006). Patients with NSAP had smaller MUP and SMUP amplitudes compared to the control and LE groups (*p *< 0.006). MUP durations were significantly longer in the NSAP, LE and at-risk groups compared to the control group (*p *< 0.006), but significantly shorter in the NSAP group compared to the at-risk and LE groups; SMUP duration was significantly shorter in the NSAP group compared to the control and at-risk groups (*p *< 0.006). NSAP and LE subjects had lower mean MU firing rates than the control subjects (*p *< 0.006).

With respect to the number of subjects used in each of the groups, having a sample size of eight for the asymptomatic at-risk group was small and lower than desired; nonetheless, there was sufficient statistical power to detect differences in many of the measures studied. Ideally, we would have had a larger sample of individuals at-risk (n = 20), but our recruitment efforts were limited particularly by our exclusion criteria that required individuals at risk of RSI to not have had any previous upper extremity signs or symptoms Our small sample size for the at-risk group was, however, in line with the literature. Greening et al. [13] found differences in longitudinal nerve movement when only seven control subjects were compared to eight subjects with NSAP (wrist flexor group), and Boe et al., [30] found differences in motor unit number estimates (MUNE) comparing only 10 healthy subjects to nine patients with amyotrophic lateral sclerosis (ALS). In a recent study that assessed fine motor control in patients with occupation-related lateral epicondylitis, the 28 subjects who participated had a mean age of 42.0 ± 6.4 years [31], which is similar to the at-risk, LE and NSAP groups (age = 44.75 ± 13.48, 50.25 ± 9.21 and 46.6 ± 10.7 years, respectively) in the current study. Our sample is consistent with the repetitive strain injury literature with respect to sample size and age.

The clinical outcome measures revealed that there was increased ECRB muscle sensitivity (PPtol), increased disability (DASH), and decreased health-related quality of life (SF-36) associated with NSAP as compared to individuals at-risk, but that individuals with NSAP were no more sensitive to pressure and no more disabled than individuals with LE. The DASH questionnaire is scored out of 100, with a higher score indicating a greater disability. The DASH scores measured from our LE group (32.39 ± 15.10) were similar to those found in a previous study of LE patients (33.0 ± 15.9) [32]. The analysis of health dimensions from the SF-36 questionnaire revealed that the pain NSAP and LE individuals experienced was related to a decreased health-related quality of life. In a study that examined physical and psychosocial workplace factors on neck/shoulder pain with pressure tenderness in the muscles of individuals performing highly repetitive, monotonous work, all eight dimensions of the SF-36 questionnaire were significantly reduced when compared to individuals without pain [33].

It has been suggested that tender points within an affected muscle are sites of local pathology, and they are believed by some to be due to local muscle spasm or fibrosis [34,35]. The physiological mechanisms underlying PPtol changes have also been suggested to reflect muscle [36] or nerve dysfunction [37,38]. Reduced PPtol has been observed in medical secretaries [39], and automobile assembly line workers [40,41]. The amount of pressure that could be tolerated over the ECRB muscle was significantly lower in our NSAP group compared to the at-risk subjects. In LE, lower pain tolerance levels have been observed in the ECRB muscle compared to the PPtol in control subjects [42]. We did not find a significant reduction in the PPtol of our subjects with LE.

Using quantitative electromyography to study shape characteristics of MUPs provide insight into the underlying pathophysiology of neuromuscular diseases [16-18]. In myopathies, MUPs have reduced durations and amplitudes due to loss of muscle fibers or fibrosis [17], and increased complexity in the MUP waveforms [17,19]. In neuropathies, increases in MUP duration, amplitude, number of turns and phases are caused by increased fiber number and density from the reinnervation process [17]. The quantitative information used in the current analysis included the amplitude, duration, area-to-amplitude ratio (AAR), and number of phases of MUPs, as well as the amplitude, area and duration of SMUPs and mean MU firing rate. In general, SMUP parameters have shown higher reliability scores than needle-detected MUP parameters [43], as they are less affected by the location of the detection surfaces [44]. The quantitative EMG analysis results indicate that there were significant differences between subjects with NSAP and individuals with LE, healthy control subjects or asymptomatic individuals exposed to similar repetitive work tasks. To our knowledge, this is the first study that has found measurable differences in electrophysiological characteristics between individuals with NSAP and healthy control subjects. The fact that differences in MUP morphology were present in a comparison between muscles affected by NSAP and muscles affected by LE suggests that these conditions are not a continuum of a single pathophysiology. In fact, comparing the morphology of the MUPs of the LE group to those of the NSAP group (increased LE MUP amplitude and AAR and increased LE SMUP amplitude, area and duration relative to NSAP values) suggests that the motor units in the ECRB muscles of the LE group subjects are larger than those of the NSAP group, which in turn suggests that at least some individuals in the LE group might have had neuropathic changes in their affected muscle. This supports the clinical belief that LE may be associated with cervical radiculopathy [45]. Although we excluded individuals with signs or symptoms of radiculopathy, quantitative EMG analysis may be more sensitive than subjective and objective clinical examination. Interestingly, the ULTT3 results were only found positive in subjects in the LE group, and this test is thought to detect compression or traction of the nerve [22].

The MUP-based electrophysiological findings suggest that NSAP may be myopathic in nature, since MUPs detected in the affected muscles of this group were smaller (lower amplitudes) and more complex (more phases) than MUPs detected in the muscles of the control group and shorter than MUPs detected in the muscles of the age-matched at-risk and LE groups [17]. In addition, all of the SMUP parameters were significantly smaller in the NSAP group, further supporting the suggestion that NSAP may be associated with changes within the muscle itself such as a loss of muscle fibers, fibrosis [17], or atrophy. The observation that the morphological parameter values of the MUPs and SMUPs for the at-risk group fall between those of the NSAP group and those of the control group suggests that repetitive work may cause morphological changes within the ECRB muscle. These changes may predispose individuals to developing painful muscles.

The sample recruited for the current study had significantly younger control subjects than the other three groups. This was related to the main criteria for fitting into the asymptomatic control group, where individuals were to be healthy and to not perform regular repetitive activities in their job or leisure activities; most of the control subjects were therefore undergraduate or graduate students who did not yet have a full-time occupation, and who did not spend more than four hours per day performing computer keyboard work. This age gap is consistent with another study where quantitative MUP analysis was used to compare control subjects (27 ± 4 years) to individuals with ALS (52 ± 12 years) [30]. However, with normal aging, muscle atrophy occurs as a result of fiber loss and the total number of fibers within a given muscle is reduced. The surviving fibers often show evidence of fiber-type grouping where denervation may have occurred [46]. McComas et al. [47], investigated the effects of aging on the number of motor units in the thenar, extensor digitorum brevis and biceps brachii muscles, and they found losses of motor units with increasing age. In the distal muscles, the declines became statistically significant in the 60 to 79 year age group, and were even more evident in the 80 to 98 year age group, whereas the numbers of motor units in the bicep brachii were well maintained in all age groups. Our NSAP subjects were close to the 60-year-old mark (50.25 ± 9.21); therefore the effects of aging on their ECRB muscles should be considered. The age of our at-risk and LE subjects was also higher than that of our control subjects and similar to that of the NSAP group. Therefore aging would likely have affected these three groups similarly.

As an example of possible aging effects, consider that the control group had significantly shorter MUP durations and significantly smaller AAR values compared to the other groups. The shorter MUP duration and smaller AAR values found in the control group are not consistent with our speculation that NSAP may be caused by a myopathic process. Decreased MUP duration and reduced AAR values are important distinguishing features of needle-detected MUPs in neuromuscular disease, and in myopathic disease the MUPs are expected to be shorter in duration and have smaller AAR values than in unaffected muscles [48]. This discrepancy in our findings may be related to the difficulty in reliably determining MUP onset and end markers [44,49-52] and the consequences of this on the MUP duration and AAR measures [43,49,51,52]. However, as individuals age, the durations and AAR values of their needle-detected MUPs increase [53,54]. This, combined with the fact that the durations and AAR values of the NSAP MUPs were shorter and smaller respectively than those of the closer age-matched LE and at-risk groups, strongly suggests that the discrepancy is more likely related to the age difference between the control and other groups.

Furthermore, although our NSAP subjects were not over the age of 60 (where significant decreases in the number of motor units within a distal muscle begins to be observed [47]), they were close to the age of 60. As such, the effects of the age of our NSAP group would be to increase MUP amplitude [54] and to increase SMUP amplitude and area [55]. Thus our findings of lower MUP amplitude and lower SMUP amplitude and area in the NSAP group relative to the control group are even more strongly suggestive of myopathic changes as opposed to motor unit loss (i.e., neuropathic changes) in the muscle of the subjects in the NSAP group.

Future research investigating motor unit number estimation (MUNE) through EMG as well as fiber type composition through histological studies in this patient population would provide further insight into the pathophysiology of NSAP.

The statistically significant reduction in firing rates seen in the LE and NSAP groups relative to the control group may not be clinically relevant, as the differences were very small (Control group = 14.98 ± 2.97 Hz, LE group = 13.86 ± 2.71 Hz, and NSAP group = 14.53 ± 2.68 Hz) and the mean firing rates in all groups remained within a normal range (5–20 pps for wrist extensor contractions between 5–20% of MVC [56]). The reduction in MU firing rates observed may also be attributed to the age differences between the groups [57].

## Conclusion

All the detected MUP size-related parameters revealed that the NSAP group had significantly smaller MUPs than the control and LE subjects. Smaller MUPs are often associated with myopathic conditions and may be indicative of fiber atrophy and/or loss within a motor unit. Evidence of these same changes was found in the at-risk subjects, whose amplitude measures were also smaller than the control subjects, but not smaller than the subjects with NSAP. As such, these findings may reflect the effects of habitual use on muscle structure (all NSAP and at-risk subjects had occupations that required repetitive low-level contractions of the ECRB muscles) and not pathology. The fact that the MUP and SMUP durations are not the same for the at-risk and NSAP subjects provides evidence that there may be muscle pathology in NSAP and that our results do not simply reflect a training effect.

Interestingly, the group of subjects with LE actually showed increases in MUP size relative to the control subjects, suggesting that the neuromuscular changes seen in individuals with NSAP are not the same as those seen in individuals with LE. The larger MUPs in the LE group are consistent with the clinical belief that patients with LE are often thought to have cervical radiculopathy. A prospective study is needed to confirm any causal relationship between smaller MUPs and SMUPs and NSAP as found in this work.

## Competing interests

The authors declare that they have no competing interests.

## Authors' contributions

KMC carried out the recruitment and testing of participants, acquisition of data, analysis and interpretation of data, and writing the manuscript. LM and DWS conceptualized the research question and study design, and provided guidance in terms of data acquisition, analysis and interpretation. LM was the senior researcher and principal investigator of the research study.
